# Landscape ecology reaching out

**DOI:** 10.1007/s10980-021-01301-y

**Published:** 2021-07-26

**Authors:** Felix Kienast, Gretchen Walters, Matthias Bürgi

**Affiliations:** 1 Swiss Federal Institute for Forest, Snow and Landscape Research WSL, CH-8903 Birmensdorf, Switzerland; 2 Faculty of Geosciences and the Environment, Institute of Geography and Sustainability, Quartier Mouline, CH-1015 Lausanne, Switzerland; 3 Department of Anthropology, University College London, Gower Street, WC1E 6BT, London, UK; 4 Institute of Geography, University of Bern, CH-3012 Bern, Switzerland


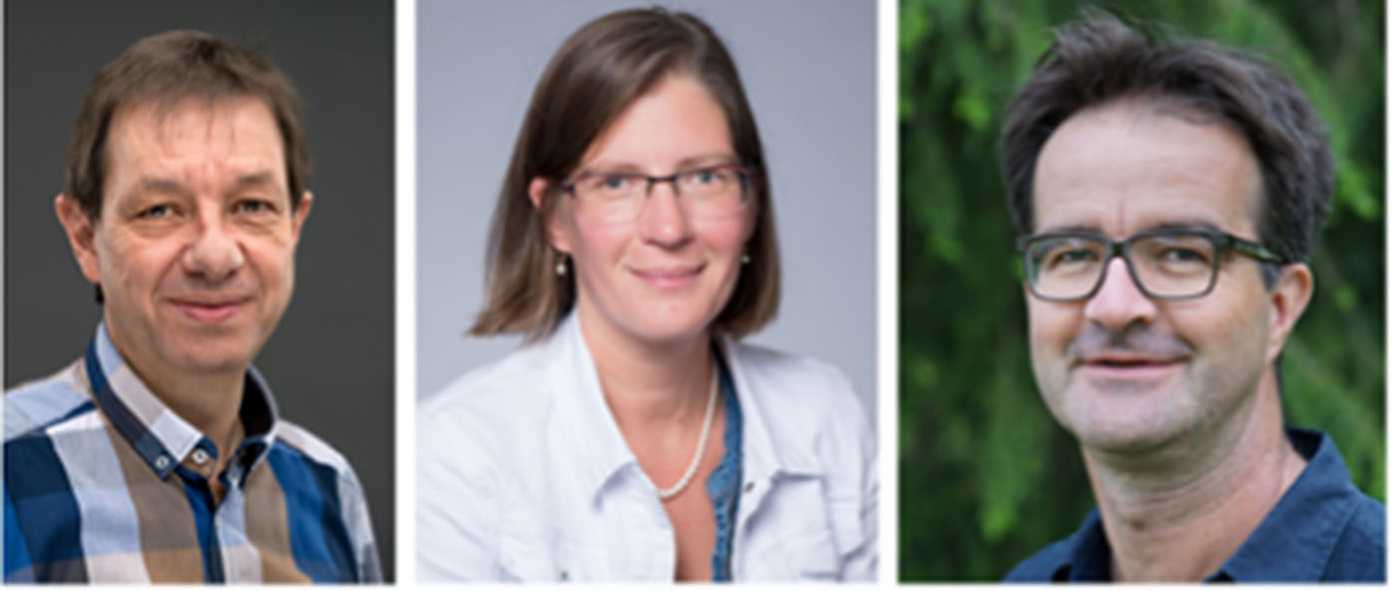


## Background of the special issue

When Troll coined the term “landscape ecology” in the 1930s (Haber 2004), the field was established as a broad, interdisciplinary field, and landscape ecologists have been collaborating intensively with researchers from neighboring fields ever since. These disciplines associated with landscape ecology have benefited strongly from the spatial concepts of landscape research and from its systemic approach. Conversely, landscape ecology has also benefitted from this interdisciplinary exchange. For example, it would never have been possible for landscape ecology to deepen sufficiently the understanding of the role of landscapes for establishing a bond with a place without collaborating with social scientists and psychologists.

In this editorial we describe important fields and technologies, which have over the course of decades contributed to landscape ecology or are still enriching the field. We refer to the resulting impetuses for the development of landscape ecology as “stimuli”. They are selected based on the authors’ broad understanding of landscape ecology. These stimuli provide a context for the contributions of this special issue, attributing them to one another of the neighboring fields. In other words, each paper helps demonstrate the inspiring interlinkages between landscape ecology and neighboring fields. Landscape ecology today encompasses a dazzling array of topics, approaches, collaborations, which on the one hand is exciting, but on the other hand raises the question of the common denominator, the common ground that distinguishes landscape ecology from other fields. What unites all these approaches as a field of study and ensures that they do not appear to be arbitrarily thrown together? We feel that the papers listed in the present special issue are suited to explore the diversity and richness of inspiring interdisciplinary collaboration, and showcase niches where landscape ecology “reaches out” while staying grounded in its rich disciplinary heritage. Under no circumstance, can the papers claim to represent the field comprehensively; nonetheless, they permit readers to explore how landscape ecology exchanges with, borrow from, and contributes to neighboring fields. Obvious gaps will be discussed in the following section—but of course the selection, as well as the references to gaps, reflect our own views and thematic positions in the broad field of landscape ecology. Had other editors compiled this special issue, other contributions would likely have been selected.

For the selection presented here, we consulted the congress proceedings of recent IALE conferences, requested contributions from our community, and received acceptances as well as rejections. COVID-19 also had a hand in this venture: some contributions had to be withdrawn due to an overload of online teaching or personal challenges. We especially regret that we were not able to receive contributions from the eminently important sister discipline landscape architecture as well as a paper illustrating the role and relevance of landscape ecology in climate change adaptation. There are traditional interlinkages such as the “pattern and process” stimulus that has become an intrinsic topic of landscape ecology but also the link with the social sciences. The latter is an example of a significant way that landscape ecology has evolved by including people as decision-makers, and so considering how those decisions transform landscapes. This inclusion, as necessitated by sustainability science, changes the way landscape ecology engages the social and even political sciences (see Wu 2021, this issue).

## A brief history of the field

We do not aim to provide a complete history of landscape ecology, as other authors have already done this (e.g. Antrop 2000). However, assessing the trajectories of how the field has developed provides a starting point to assess the origins of the stimuli and to understand what role they played. We are well aware that the trajectories presented here are biased towards the Anglo-European notions of landscape and landscape research, a bias that we attempt to correct in the outlook of the editorial.

It is generally accepted that prior to the Enlightenment (Europe, 17-18th centuries), the term landscape, or its predecessors, was more related to “cultivated land” in Haber's sense (Haber 2004). This meaning of landscape is still quite frequently found, for example, in conversations with farmers in Europe and elsewhere. Before the Enlightenment, nature and wilderness were considered a threat by Europeans, with the aesthetically pleasing landscape being a product of elite philosophers during the period. Simultaneously these European landscapes of rural areas and the rural life were glorified, as we read in, e.g., Albrecht von Haller’s (1708–1777) “The Alps” (Haller et al. 2019). The European landscape was domesticated in gardens and painted in picturesque ways during the Romantic Era (end of 18th to the nineteenth century). The first European scientific impetus for landscape research came from Alexander von Humboldt, who laid the foundation for landscape science during his round-the-world travels (Haber 2004). His landscape science branched out into the disciplines of geography, botany, and chemistry. However, it took almost 100 years for geography and ecology to merge again in Troll’s concept of Landscape ecology.

Landscape architecture has a more linear, and less interrupted history. Inspired by the idea of the European landscape garden of the Enlightenment, emerging cities and wealthy individuals started to finance urban and mansion parks. It was merchant Gilbert Laing Meason who coined the term Landscape Architecture in his book “On The Landscape Architecture of the Great Painters of Italy”, published in 1828 (Meason 1828). Thanks to Scottish horticulturist John Claudius Loudon, the term Landscape Architecture was promoted by the first professional landscape architect Frederick Law Olmsted, the designer of New York City’s Central Park and the Parc du Mont-Royal in Canada. Since 1860, the profession of landscape architecture officially existed. By designing parks and supervising the work to be carried out, landscape architecture was—from the beginning—a pragmatic and practice-oriented branch of landscape science, originating before landscape ecology. However, it took until the early 2000s for landscape ecology to proactively recognize “design” as an important pillar of the field (Nassauer and Opdam 2008). Since then, landscape architecture and landscape ecology have converged in many countries around the world.

The time period from 1860 is characterized by a departure from the representation of the picturesque landscape of the European Romantic Era. European Impressionism and New Realism discovered the landscape as an “outdoor studio”, as Monet often called it. All types and elements of landscapes were depicted, the picturesque rural life, as well as the industry and urban infrastructure. Worth mentioning in the context of the emerging landscape science is the American Transcendentalist School in the first half of the nineteenth century that highlighted the goodness of humanity and the glories of nature. Prominent representatives were Americans Ralph Waldo Emerson and Henry David Thoreau. Although these thinkers made valuable contributions to the debate on landscape, there was also an uptake of the idea of the "uninhabited wilderness" from the cultivated land, and most notably by Scottish-American naturalist John Muir. Along with other key proponents of the wilderness idea, they proposed a nature-culture dualism (Cronon 1996), resulting in the creation of the American National Parks model in the 1870s, which evicted many native Americans from their territories (Spence 1996). This model was unfortunately exported to many countries (Adams 2004), resulting in the creation of strict protected areas which resulted in dispossessing many people from their lands (Brockington and Igoe 2006). The discourse of “pristine” wilderness (Wuerthner et al. 2015) is associated with great injustices and land dispossession of local populations and the loss of land and natural resource rights, a fact that is only now slowly being addressed (Gilio-Whitaker 2019). Although many scientists now see wilderness as an outmoded concept, it is still prevalent in conservation thinking to the present day (Fernández-Llamazares et al. 2020), including in the current “half-earth” debate (Büscher et al. 2017; Wilson 2017).

## The stimuli

The previous section sought to place the development of landscape research in its wider cultural context. In this section, we zoom in on the scientific context by describing some important scientific stimuli in the development of landscape ecology. Most of the stimuli, of course, work in both directions, to different degrees: the neighboring field is inspired by the exchange with landscape ecology and landscape ecology is inspired by the neighboring field. For each stimulus, we introduce the relevant papers included in this special issue.

### Introducing new technologies to facilitate a view of the landscape from above

The first important breakthrough for scientific landscape ecology was the wall-to-wall possibilities of aerial photography. This pattern of a technologically-driven interdisciplinary field shapes the development of landscape ecology and did not end with aerial photography. In the 1970s and 1980s, landscape ecology underwent a quantitative phase triggered by the development of remote sensing, Geographical Information Systems, spatial modeling and quantitative pattern analysis. As shown in the paper of Pazur et al. (2021), we are in a similar phase, where the availability of novel, temporally and spatially finely resolved remote sensing data, combined with drastically increased computational capacities fuel the vision of achieving wall-to-wall coverage of landscape ecological pattern and processes. The ultimate goal of this type of research is to reach out to land use planning practice and deliver environmental data to guide sustainable agriculture and forestry, while also providing biodiversity-relevant high-resolution data over large areas. An example of the latter is the paper by Dou et al. (2021) which proposes a spatially explicit model on a 1 km grid for the whole of Europe to estimate the impact of land cover and land use intensities on biodiversity, a topic that has been consistently neglected in species distribution modeling to date, even though land use is one of the most important drivers of biodiversity loss.

### Exploring the pattern and process paradigm to understand movement of biota across scales

A milestone for modern landscape ecology and an ongoing source for a fruitful scientific discourse are the stimuli coming from island biogeography and the metapopulation theories. They led to recognizing species movement in space, a topic to which North American landscape ecology has devoted itself intensively over decades and has made great contributions to spatial ecology. A logical continuation of the pattern and process paradigm is the pioneering landscape genetics work (see special issue in Landscape Ecology by Holderegger and Wagner 2006). Not only was the genetic distance between populations explained by in-between landscape properties, landscape genetics could, for the first time, confirm (and in certain cases reject) the connectivity paradigm—one of the cornerstones of practical conservation. Closely linked to landscape genetics is the field of road ecology and the corresponding fragmentation analyses of landscapes, which gained practical relevance for the planning of roads (Jaeger 2000). In our special issue the article by Jeanneret et al. (2021) is dedicated to the paradigm “patterns and processes”. The authors highlight how the spatio-temporal pattern of semi-natural elements and agricultural fields can be understood quantitatively to control pollinators and pests. But the article goes beyond understanding the processes. It has a strong transdisciplinary component and culminates in a call for promoting agroecological practices beyond the individual farm patches, using a bottom-up approach starting from agroecological lighthouse farms to farm networks encompassing entire regions.

The idea of up-scaling is picked up in the article by Garcia-Martin et al. (2020). They link the individual farm and product to the level of distant consumers in an attempt to study how global trade dynamics affect the sustainability of agricultural landscapes from which products are sourced. They focus on food products that link global consumers to production landscapes (e.g., wine from the Douro Valley) and analyze value chains to identify the environmental footprint of consumption of internationally traded products. Also heavily influenced by the pattern and process paradigm but also a good example of how remote sensing data can be used is the article by Li et al. (2021). The authors use a time series of NDVI data as a proxy for grassland productivity. The latter is then used to analyze a regime shift in a Tibetan rangeland where changing grazing patterns of yaks degrade the grasslands and make it necessary to advocate for adaptive management schemes. The study by Li et al. (2021) also highlights the importance of considering historical sources in assessing the current landscape condition, a stimulus that is discussed in the next paragraph.

### Addressing history to explore the temporal dimension of landscapes

The insight that landscape pattern and processes change dynamically over time was an important stimulus in the development of landscape research. In England, it led to prominent publications such as the book “The making of the English Landscape” by Hoskins (2006) and “The history of the countryside” by Rackham (1986). In the journal Landscape Ecology, a series of influential papers on landscape history have been published since the 1990s, starting with a methodological contribution on novel possibilities offered by GIS for analyzing historical changes in landscape pattern (Kienast 1993). GIS greatly facilitated the analysis of landscape change using time series of aerial photographs and topographic maps, and resulted in various studies on changes of pattern in landscapes (e.g. Rhemtulla et al. 2007), but also grasslands (e.g. Pärtel et al. 1999), forests (e.g. Moreira et al. 2001), or the urban fabric (Zhao et al. 2015). Apart from these core sources for geographers, other source types, such as archaeological records (Silbernagel et al. 1997), written sources, including survey records (e.g. White and Mladenoff 1994) were used, contributing to an increasingly interdisciplinary perspective on the dynamics of landscape change (e.g. Casazza et al. 2021).

Landscape archaeology is a prime example, where landscape ecological concepts provide stimuli for neighboring fields. Arikan et al. (2020) illustrate this by performing agent-based modelling for an archaeological site in Arslantepe, eastern Anatolia, Turkey. The deep-time perspective from archaeology illustrates how far back in time the human imprint in the land reaches, challenging simplistic notions of wilderness as well as of reference conditions for restoration. Tappeiner et al. (2020) propose that present patterns and processes are shaped not only by present conditions but are in various ways influenced by patterns and processes of the past. Moreover, including history in landscape ecology has to go beyond interpreting pattern and processes in their historical dimension, as this would neglect the inherent dynamics of landscape-society interactions. Therefore, the authors propose to explicitly consider pathways, a concept coming from historical sociology.

### Addressing the landscape concept in spatial planning

Since the 1980s we find an increased interest to incorporate landscape aspects into spatial planning (Leitão and Ahern 2002; Milovanović et al. 2020). This approach was motivated by the fact that knowledge about landscape quality relevant to humans, plants and animals should be part of well-informed planning documents and so guide the planning discourse. Hersperger et al. (2021) identify, based on a literature review, the landscape ecological concepts that are most often used to support landscape planning. They observe a frequent inclusion of concepts such as structure, function, change, scale etc. in landscape analyses, but less so in the context of goal establishment and monitoring. Relevant against the background of a growing planning discourse is also the paper by Wartmann et al. (2021a) on tranquility landscapes. Using social media data from Geograph UK, georeferenced user-generated landscape descriptions were filtered using keywords related to tranquility. Subsequently, an attempt was made to statistically link the dominant land use with the mention of tranquility. For water, views, and natural land use classes, people mentioned tranquility items more often, while urban land uses prompted fewer tranquility items. They conclude that such models are extremely useful for planning recreation landscapes.

### Introducing the space—place concept to interpret landscapes as social constructs

Landscapes have been shaped by physical forces, the production of ecosystem services and cultural values (Bürgi et al. 2015; Kienast et al. 2018). Troll’s vision to generate a unified ecoscience where social and physical properties of landscapes are jointly analyzed was largely dormant until the 1980s and 1990s until landscape perception and aesthetic studies became prominent in landscape ecology. However, many of these studies remained in the place-dependency mode: they described the degree to which the practical needs of people or aesthetic aspects are satisfied in a particular place. In the 1990s, Twigger-Ross and Uzzell (1996) proposed their “place-referent continuity” concepts that deal with identity-forming aspects of landscapes. Identity forms when tangible elements of the landscape are assigned specific meanings or shared values by society or social groups (Devine-Wright and Howes 2010), providing individual mental self-regulation. Thanks to the work of Hunziker et al. (2007), the aforementioned fragmented concepts dealing with the human-landscape interaction became unified in the widely cited space-place theory, a milestone in interpreting landscapes as social constructs. For this stimulus, the paper of Wartmann et al. (2021b) is a novel contribution to analyzing the perception patterns of people. It statistically analyzes how both landscape composition and social science measures contribute to explaining people’s perception and assessments of landscapes. Among other results they found that the more an area was sprawled and fragmented, the less people were satisfied with the everyday landscape. In contrast, the more people perceived landscape quality positively, the more their place attachment and satisfaction with the every-day landscape of their municipality increased.

### Including the landscape approach to co-design landscapes

A decade ago, a new stimulus for Landscape Ecology emerged under the name “landscape approach”. Although landscape level research in conservation had been known at least since the 1980s (Noss 1983), the approach saw a large uptake in conservation after the publication of the principles of applying the landscape approach (Sayer et al. 2013). The intention of the landscape approach is not fundamentally different from that proposed by the International Association for Landscape Ecology and the European Landscape Convention (ELC). What is new and worth calling it a further stimulus to landscape ecology, is the idea of co-designing with stakeholders, the clear structuring of the project agenda, and the emphasis on environmental justice and governance. Four contributions to this special issue focus on the landscape approach, which is used by many conservation organizations to foster landscape sustainability. The contribution by Reed et al. (2021) proposes that integrated landscape approaches have evolved towards the social sciences. The authors propose a reintegration of ecology into these approaches, while aiming to remain balanced with participatory stakeholder engagement. We then have two contributions which situate the landscape approach in Asia and Africa. First, we go to Indonesia where the contribution by Riggs et al. (2021) provides an in-depth view of the landscape approach used in eight conservation landscapes. Insights on the contribution of the landscape approach emerge from a series of landscape practitioner workshops, providing a clear way that landscape ecologists can engage with and inform practice, policy, and landscape sustainability. We then move to Central Africa, where the contribution by Walters et al. (2021) focuses on landscape ecology and the contribution to landscape sustainability. Two cases demonstrate landscape-scale approaches that engage conservation practitioners and conservation scientists within large-scale conservation landscapes in the Congo Basin. The theme of understanding landscape history, and cultural ways of viewing landscapes reemerges as important, as does the need for long-term collaborations of researchers in these landscapes. Taking a look at the restoration movement, the contribution by Mansourian (2021) focuses on the influence of landscape ecology and the emerging field of practice of forest landscape restoration (FLR). She shows the interrelationship between FLR and landscape ecology, including points of convergence and divergence, and questions the future of FLR as it evolves from practice to potentially a research field itself.

Landscape sustainability emerged as a key research priority in 2002 for landscape ecology (Wu and Hobbs 2002) and is defined as ‘‘the capacity of a landscape to consistently provide long-term, landscape-specific ecosystem services essential for maintaining and improving human wellbeing in a regional context and despite environmental and sociocultural changes’’ (Wu 2013). Landscape ecology has progressively sought to increase linkages to the social sciences and decision-making at different scales (Angelstam et al. 2019). Although landscape approaches are one contribution to landscape sustainability, they are not the only ones. In the final contribution of this special issue by Jianguo (Jingle) Wu (2021)**,** he proposes core questions and key approaches to landscape sustainability science. Using a cross-disciplinary approach, he proposes an updated Landscape Sustainability Science (LSS) framework, with an enhanced focus on landscape governance and institutions and local and Indigenous knowledge, a cyclical research process articulated with action, and linkages between pattern and process and drivers of change. LSS itself integrates many fields from landscape ecology to land system science, food-energy-water nexus, amongst others, and so demonstrates how landscape ecology continues to reach out and be part of collaborations with neighboring disciplines.

## Outlook—emerging potential stimuli for landscape ecology

This special issue concerns how landscape ecology has reached out and influenced other fields and vice versa. Given the diversity of disciplines involved, what does this mean for the future of landscape ecology? Will landscape ecology continue to evolve as a broad field in itself? Does it have core methods and approaches or is it changing to more inter-disciplinary and transdisciplinary approaches, such as those required by sustainability science? Regardless of the answer, the current dynamics of the field presents challenges for researchers, who may need to identify with a particular discipline early in their career paths (Bühler et al. 2006). But what we see happening in landscape ecology, is not unique. Innovation in a field often comes from the boundaries, and is influenced by how it interacts with other disciplines; it can have a core concept, theory and method, but then “dialogue with other disciplines” as it evolves (Darbellay 2015). As landscape ecology dialogues with other fields, the landscape can become a boundary object, linking the environmental and social sciences through ecosystems, place and politics of scale (Arts et al. 2017). With the landscape as a key focus, using spatial, technological, historical and social methods, the field can remain focused, yet also expand, and make practical contributions to sustainability science, conservation, land use planning, amongst others.

Given how vibrant the field is, new stimuli will continue to appear and shape its future. To conclude this editorial, we list a few of them and hope to inspire the reader to think, based on their own individual expertise, about approaches which might result in future stimuli.

### Integrating landscape governance to enable sustainable solutions in practice

As pointed out by Wu (2021) the topic of landscape governance (Görg 2007) is core to landscape sustainability science. We agree that the field still has a long way to go, to truly embrace this dimension. Although integrated landscape approaches involve addressing conflicts; at the same time, landscape governance involves decision-making processes. Both of these themes are related to the politics of landscapes, which is often the purview of political ecology and political geography. In political ecology, another domain which reaches out to many disciplines (Robbins 2012), many authors study the politics of decision-making and conflicts in landscapes, such as conservation landscapes (Clay 2016; e.g. Bluwstein and Lund 2018). However, political ecology has also been suggested to diverge away from ecology itself (Walker 2005). A fruitful engagement could be envisioned between landscape ecology and political ecology in the future, bringing ecological and social theory together at the landscape scale to inform land sustainability science.

### Looking beyond Anglo-European landscape notions to address the diversity of landscape dimensions

For a long time, landscape ecology viewed the emergence of the concept of landscape primarily from an Anglo-European perspective (Antrop 2000). This view dominated the concept and saw it confirmed by centuries of development in Anglo-European art, (garden) culture and science, as noted in the previous sections. The worldwide dominance of this view left little room for other conceptions of landscape, and it continues to occupy international agendas, despite not always having congruent concepts in many parts of the world (Gauché 2015). Corner (1999) argues that—whilst there is a sort of an environmental perception in every culture—the holistic visual landscape as known in Anglo-European culture is by far not the only way to perceive landscapes. As shown by Murton (2011), landscape concerns more than a visual representation, and is complemented by speech and sound as exemplified by e.g. the Maori in New Zealand. Today, this bias towards visual representation is increasingly being corrected by, for example, linguistic work (Mark et al. 2011), research on Indigenous knowledge, and advances in participatory GIS (e.g. Fagerholm and Käyhkö 2009). Thus, landscape perceptions are now increasingly understood in a global context.

Inspired by the assessments of Olwig (1996), Bigell and Chang (2014) and Mark et al. (2011), we suggest multiple dimensions on how landscapes are perceived, described, and experienced, e.g. a visual-descriptive dimension, a territorial dimension referring to land rights, a land use-oriented dimension with strong links to nature and ecology, and an ancestral dimension. Interestingly, many cultures around the world use one or more of these dimensions, e.g., the Tlingits of Southeast Alaska who make their living in coastal waters (Thornton 2017), or the Inuit of the George River estuaries who have developed a spatially explicit mental map of their hunting grounds using centuries-old stories and narratives and a vast array of toponyms that accurately describe horizontal and vertical units of the land–water interface. An example of the land-use-oriented dimension is given by Haber (2004), who shows how the term landscape refers to land that has been shaped by human use. Territorial associations of landscape are not only widespread in Anglo-European cultures but also elsewhere. For example, in Australia, Aboriginal peoples view “country” as a piece of land occupied by a particular group with its unique land use needs and further justified by rights and responsibilities of the ancestors (Mark et al. 2011). For ancestral dimensions of landscapes, we can point to one example amongst many, of the peoples in Central Africa who govern and manage their landscapes through cultural practices which honor ancestors and land spirits, often in specific forests, rock outcrops or water sources, as is the case of the Pové and Batéké peoples of Gabon (Walters et al. 2015).

At the beginning of this editorial, we raised the question of what unites the very heterogeneous approaches of landscape ecology and ensures that the field has a common denominator. After editing this special issue, we have come to the conclusion that the best common denominator of the field is indeed the notion “landscape”, fundamentally similar on a global scale, but interpreted in region-and disciplinary-specific ways. This pluralistic interpretation of “landscape” has shaped landscape ecology for decades and has ensured an open and democratic debate about what landscapes are and mean for ecology and society alike. Considering the broad array of contributions in the current special issue, we are convinced that this debate will continue to stimulate both research and knowledge exchange between research, practice and society. “Landscape” seems to be the appropriate dimension at the right scale. It enables innovative research, and it touches people in their daily lives, helping them to perceive possibilities for future development.
